# Efficacy, Benefits, and Harms of a Self-management App in a Swedish Trauma-Exposed Community Sample (PTSD Coach): Randomized Controlled Trial

**DOI:** 10.2196/31419

**Published:** 2022-03-30

**Authors:** Ida Hensler, Josefin Sveen, Martin Cernvall, Filip K Arnberg

**Affiliations:** 1 National Centre for Disaster Psychiatry Department of Medical Sciences Uppsala University Uppsala Sweden; 2 Psychiatry Department of Medical Sciences Uppsala University Uppsala Sweden

**Keywords:** PTSD, self-management app, mHealth, RCT, negative effects, mobile phone

## Abstract

**Background:**

Self-guided interventions may complement and overcome obstacles to in-person treatment options. The efficacy of app interventions targeting posttraumatic stress disorder (PTSD) is unclear, and results from previous studies on PTSD Coach—an app for managing trauma-related distress—are inconsistent.

**Objective:**

This study investigates whether access to the Swedish version of the PTSD Coach affects posttraumatic stress, depressive, and somatic symptoms. In addition, we aim to assess the perceived helpfulness, satisfaction, negative effects, response, and remission related to PTSD Coach.

**Methods:**

Adults who had experienced potentially traumatic events in the past 2 years were randomized (1:1) to have access to PTSD Coach (n=89) or be on the waitlist (n=90). We assessed clinical characteristics at baseline (semistructured interviews and self-rating scales) and after 3 months (self-rating scales). We analyzed the data in R software using linear mixed effects models, chi-square tests, and Fisher exact test.

**Results:**

Intention-to-treat analyses indicated that access to PTSD Coach decreased posttraumatic stress and depressive symptoms but not somatic symptoms. More participants who had access to PTSD Coach responded with clinically significant improvement and fewer instances of probable PTSD after 3 months compared with waitlist controls. Overall, participants found that PTSD Coach was slightly to moderately helpful and moderately satisfactory. Half of the intervention group (36/71, 51%) reported at least one negative reaction related to using PTSD Coach (eg, disappointment with the app or its results, arousal of stress, or distressing memories).

**Conclusions:**

Using PTSD Coach may trigger symptoms among a few users; however, most of them perceived PTSD Coach as helpful and satisfactory. This study showed that having access to PTSD Coach helped improve psychological trauma-related symptoms. In addition, we have discussed implications for future research and clinical practice.

**Trial Registration:**

ClinicalTrials.gov NCT04094922; https://clinicaltrials.gov/ct2/show/NCT04094922

## Introduction

### Background

Psychological trauma, often recognized as posttraumatic stress disorder (PTSD), is an important public health problem. Most of the world’s population will be exposed to one or more traumas throughout their lifetimes [1,2]. The lifetime prevalence of PTSD is nearly 4%, and it is associated with considerable burden [2]. A substantial impairment is also noted among people who experience posttraumatic stress symptoms but do not meet the complete diagnostic criteria for PTSD [3]. Trauma survivors experience significant personal and structural barriers to seeking help, including stigma, time and resource constraints, and lack of knowledge and access to mental health care [4].

PTSD Coach is a self-management mobile app for improving knowledge of PTSD symptoms and providing coping strategies for trauma-related acute distress [5-7]. The app provides psychoeducation about the effects of trauma, a self-rating scale for posttraumatic stress, and contact information to reach professional help and support organizations. It also contains a database of self-guided exercises inspired by cognitive behavioral treatment methods such as mindfulness, stress reduction techniques, grounding, positive psychology, and cognitive restructuring [6,7]. The efficacy of app interventions that target PTSD is unclear [8]. Self-guided interventions such as PTSD Coach could not replace, but may complement, in-person treatment options [9] as stand-alone interventions or as additions to psychological or medical treatments.

PTSD Coach has shown promise as a beneficial intervention in Western countries [7]. However, the results from uncontrolled studies regarding PTSD Coach and a decrease in posttraumatic stress or depressive symptoms are inconsistent [10-12]. The results from prior randomized controlled trial (RCT) studies [6,13,14] of PTSD Coach also differ, perhaps owing to differences in the operationalization of outcomes and sample sizes. For example, using PTSD Coach with or without clinician support decreased PTSD symptoms but did not change depressive symptoms [14], and having access to PTSD Coach was related to greater improvements in PTSD symptom severity [6,13] and depressive symptoms compared with controls [6]. However, the results were inconclusive, as symptoms after the intervention did not differ compared with controls [6,13].

Users tend to like PTSD Coach. Although some disagree [13], the app was generally considered moderately to extremely helpful in US samples [5,15] and slightly to moderately helpful in a pilot study of the Swedish app [12]. Users endorse being moderately to extremely satisfied [5,15] or slightly to moderately satisfied with the app [12]. Overall satisfaction seems to be higher among smartphone owners than others, whereas perceived helpfulness do not differ [5]. Similarly, users have expressed that previous digital skills may benefit the use of PTSD Coach [16]. In addition, some users questioned whether using PTSD Coach without clinician support could be harmful [16]. Deterioration (ie, worsening of symptoms [17]) should be considered a side effect if the intervention cannot be ruled out as a probable cause [18]. To the best of our knowledge, no investigation of PTSD Coach has reported the presence or absence of deteriorated symptoms [6,10,11,13,14]. Other possible negative effects are treatment-emergent reactions (referred to in this paper as negative reactions, ie, unwanted reactions) instigated by the use of an intervention [17]. Most researchers do not report the presence or absence of negative reactions to PTSD Coach [6,10,11,13,15]. Negative reactions such as negative emotions, psychological symptoms [16,19], and unfulfilled expectations [12] in response to content or technical issues have been reported in focus groups, interviews, and reviews on the web.

To summarize, the efficacy, benefits, and risks of self-management interventions for posttraumatic stress should be evaluated [8], especially if they are intended to be distributed without clinical support. The Swedish version of PTSD Coach has yet to be evaluated in an RCT. Sweden is a relatively sparsely populated country with high levels of smartphone use. Mental health apps may therefore be particularly well-suited for use as a complement to existing services. In addition, although exposure to potentially traumatic events and posttraumatic stress are associated with somatic symptoms and disease [20] and are subject to improvement with standard therapies such as medication or cognitive behavioral therapy [21], somatic symptoms have not been investigated concerning the use of PTSD Coach.

### Study Aims

We investigated whether access to PTSD Coach affected symptoms of posttraumatic stress (primary outcome), depression, and somatic illness (secondary outcomes) in an RCT. We also conducted post hoc analyses to explore response rates, clinically significant change, and deterioration in posttraumatic stress symptoms and remission rates of probable PTSD. Finally, we investigated perceived helpfulness, satisfaction, and negative reactions associated with PTSD Coach.

## Methods

### Design

We conducted an RCT with a parallel group, mixed model design to compare a self-management intervention with a waitlist. The intervention group (app access) had access to PTSD Coach, and the waitlist group (waitlist) did not have access to PTSD Coach for 3 months.

### Participants

Adults (aged ≥18 years) who resided in Sweden with Swedish verbal and written comprehension and ownership of a smartphone were eligible to be included in the study. Additional inclusion criteria were exposure to a potentially traumatic event in the past 2 years, according to the Diagnostic and Statistical Manual of Mental Disorders, Fifth Edition (DSM-5) criteria, and mild to severe posttraumatic stress symptoms (PTSD Checklist for DSM-5 total score ≥10). Exclusion criteria included potentially life-threatening or harmful living conditions or symptoms (eg, recurring or ongoing traumatic event exposure, severe suicidal plans or ideation, current alcohol or drug abuse, lifetime manic or hypomanic episodes, or psychotic episodes). Additional exclusion criteria were current or pending psychotherapy, medical treatment changes, and medication with counterindications such as benzodiazepines. Minor adjustments were made after the trial commenced. Participants who screened positive for alcohol or substance abuse in early remission (<12 months) with the current treatment were accepted in the study.

The required sample size of 160 participants was determined by an a priori power analysis in G*Power (version 3.1; Heinrich-Heine-Universität Düsseldorf) [22] based on the effect size of Cohen *d*=0.5 in the pilot study [12] and anticipated attrition of up to 25%.

### Procedures

Enrollment began in May 2019 and ended in June 2020. We collected data nationwide in Sweden from Uppsala University. Study data and email invitations were managed using REDCap (Research Electronic Data Capture; [23]) hosted at Uppsala University.

Potential participants were recruited through social media advertisements linked to a web-based screening questionnaire. Eligible participants provided an email, received the consent form, and provided written informed consent. We informed participants how their data were managed and that participation was voluntary and confidential. Consenting participants booked an appointment for a phone interview with a member of the research team to confirm eligibility and assess psychiatric symptoms.

Subsequently, participants completed a baseline questionnaire before randomization. Participants randomized to access to the app were emailed written instructions for downloading PTSD Coach and instructed to use the app as they pleased. Participants who requested further guidance were encouraged to explore the app to identify helpful content. Participants on the waitlist received written notice through email that they would gain access to the app after the first follow-up assessment.

We called all participants and offered the opportunity to ask questions regarding the study or technical support, 7 days after randomization. All participants responded to daily assessments for 21 days in a separate investigation [24] and received a follow-up questionnaire with the primary and secondary outcomes 3 months later. Participants with access to the app responded to additional questions regarding helpfulness, satisfaction, and negative reactions. Participants were compensated with gift cards to the cinema after completing the follow-up questionnaire. Participants who left the study or were excluded before the follow-up assessment received a gift card.

### Materials

#### The Intervention

The mobile app PTSD Coach was developed by the Veterans’ Affairs National Center for PTSD and the Department of Defense’s DHA Connected Health [5,25]. The resources in PTSD Coach are divided into four sections: *learn* (psychoeducation about posttraumatic stress, treatment, and coping in families), *track* (symptom self-evaluation with rating history and automatic feedback), *manage symptoms* (exercises for distress management inspired by cognitive behavioral therapy), and *get support* (contact information for crisis resources, professional assessment and treatment, and platforms and advice promoting social support). Self-guided exercises in the app are prompted by voice, video, or text.

The Swedish version [12] was translated and adapted from the American original to a Swedish civilian context [5,6,13]. A team of 6 clinical psychologists, researchers, and psychology students conducted the first translation for the pilot trial of the app [12], and the authors revised the adaptation based on a version update of the original and input from Swedish users. During this trial, the Swedish version of PTSD Coach was not publicly available on app stores; therefore, access to the app could be restricted to participants with access to the app. However, the American version of PTSD Coach was available on app stores.

#### Posttraumatic Stress

The primary outcome, posttraumatic stress, was assessed at screening, baseline, and follow-up with the Posttraumatic Stress Disorder Checklist for the Diagnostic and Statistical Manual of Mental Disorders, Fifth Edition (PCL-5) [26] in Swedish [27]. The PCL-5 is a 20-item self-report measure that maps directly onto the DSM-5 PTSD symptoms. The items are rated on a 5-point scale ranging from 0 (*not at all*) to 4 (*extremely*) [26], yielding a total sum of 0 to 80. The Swedish version was developed using a standard back-translation process [28] and corresponds well to the gold standard assessment of PTSD symptoms [27]. A total sum of ≥31 to 33 points may indicate probable PTSD in both the original and Swedish versions [27,29]. A ≥10-point difference in the total sum score on previous versions of the PTSD Checklist has been estimated to equate with clinically significant change [6,13,14,30], and the range for clinically significant change is presumably similar for PCL-5 [29].

#### Depressive Symptoms

The secondary outcome depressive symptoms was assessed at baseline and follow-up with the Swedish version [31] of the Patient Health Questionnaire (PHQ)-9 [32]. The questionnaire is a widely used 9-item self-report measure of the DSM-5 criteria for depression with an additional item assessing functional impairment. The suggested cut-offs for probable depression range from 9 to 12 points [32,33]. The Swedish version has demonstrated satisfactory psychometric properties [31].

#### Somatic Symptoms

The secondary outcome, somatic symptoms, was assessed at baseline and follow-up with the Swedish version [34] of the PHQ-15 [35]. The self-rating scale consists of 15 items that measure the most common somatic symptoms reported in primary health care. The Swedish version of the PHQ-15 has favorable psychometric properties for the quantification of somatization in Swedish and similar populations, with suggested cut-off scores for symptom severity at 0 to 4 indicating minimal, 5 to 9 indicating low, 10 to 14 indicating moderate, and ≥15 indicating high symptom severity [34].

#### Helpfulness, Satisfaction, and Negative Effects

Perceived helpfulness and overall satisfaction with PTSD Coach were measured with a Swedish version [12] of the PTSD Coach survey [5] at follow-up. The survey includes 14 questions that assess the perceived helpfulness of the app and 1 item asking about user satisfaction. Multimedia Appendix 1 provides all items. Negative reactions were measured using the Negative Effects Questionnaire [36]. The original version was developed in Sweden and is available in several languages to assess the negative effects of psychological interventions. In this study, references to therapists and treatment in the items were changed to the app. Respondents rated whether they had experienced 20 potential negative reactions during the period they had access to PTSD Coach, how negative reactions affected them, and the probable cause of the reaction (ie, “other circumstances” or “used the app”). Multimedia Appendix 2 shows all items.

#### Demographic and Clinical Characteristics

During the phone interview, the interviewer explored exposure to potentially traumatic events with open-ended questions (eg, “Please briefly tell me about the kind of severe event that you experienced at that time” and “What happened? You don’t need to tell me everything; I just need to understand what kind of event it was”) and categorized answers into categories from the Life Events Checklist for DSM-5 [37] in Swedish [27]. We assessed psychiatric comorbidity during the baseline phone interview with the Mini International Neuropsychiatric Interview [38] (Swedish version 7.0.0). We asked questions regarding demographic information, previous smartphone use, and previous treatment in the baseline questionnaire. Furthermore, participants provided information regarding treatment changes, the use of PTSD Coach, or other self-management apps in the follow-up questionnaire.

### Randomization

An external statistician generated the allocation sequence in R software (R Foundation for Statistical Computing; Multimedia Appendix 3): a random number table with equal allocation to access PTSD Coach or waitlist (1:1) with an unstratified block design fixed at 20 allocations. The first author (IH) uploaded the random number table without reviewing it into the Uppsala University REDCap randomization tool before data collection. The block size, R-script, and allocation sequence were concealed to members of the research team who enrolled participants (the first author, a clinical psychologist, and 2 psychology students) or randomized participants (the first author, a PhD student, and a clinical psychologist). The condition was revealed in the REDCap graphical user interface, nonblinded but unalterable, after randomization.

### Data Analysis

We analyzed data in R (versions 3.5.1 and 4.0.5) using linear mixed effects models (*nlme* package v3.1-141). We specified 3 separate multiple regression models a priori with the direct and interaction effects of condition and time on posttraumatic stress, depressive symptoms, and somatic symptoms. All randomized participants were included in the intention-to-treat and per-protocol analyses. We replaced single missing item ratings in the outcomes with the individual’s mean item rating (n=24 had 1 missing item for the PHQ-15 at baseline, and n*=*1 had 1 missing value for the PHQ-15 post intervention). Missing data for the outcomes at follow-up were assumed to be missing at random conditional on the baseline PCL-5 scores, as dropout was related to the baseline PCL-5 scores but not to any other baseline variables. We addressed the missing data using multiple imputations in the primary analyses, and we conducted sensitivity analyses, as reported in Multimedia Appendix 4. We included baseline posttraumatic stress as a predictor and imputed each outcome in 500 data sets (10 iterations) with predictive mean matching [39] (*mice* package v3.13.0 and *miceadds* package v3.11-6). We report pooled parameter estimates across all imputations. We calculated model-based, between-group standardized mean differences (Cohen *d*) with 95% CIs on the pooled within-group SD at baseline [40]. Negative reactions that were caused by circumstances other than using PTSD Coach were recorded as 0 (absent).

We conducted post hoc tests to assess whether the number of participants who reported clinically significant improvement or deterioration in posttraumatic stress (a 10-point difference on the PCL-5 from baseline to follow-up) or screened positive for PTSD (≥31 points on the PCL-5) at baseline and follow-up differed between conditions using chi-square tests. In addition, we explored whether remission from probable PTSD (≥31 to <31 points) or development of probable PTSD (from <31 to ≥31 points) differed between conditions using chi-square and Fisher exact tests.

### Ethics Approval

The regional ethical review board in Uppsala, Sweden, approved the study procedures before data collection (reference 2018/319).

## Results

### Overview

**Figure 1:**
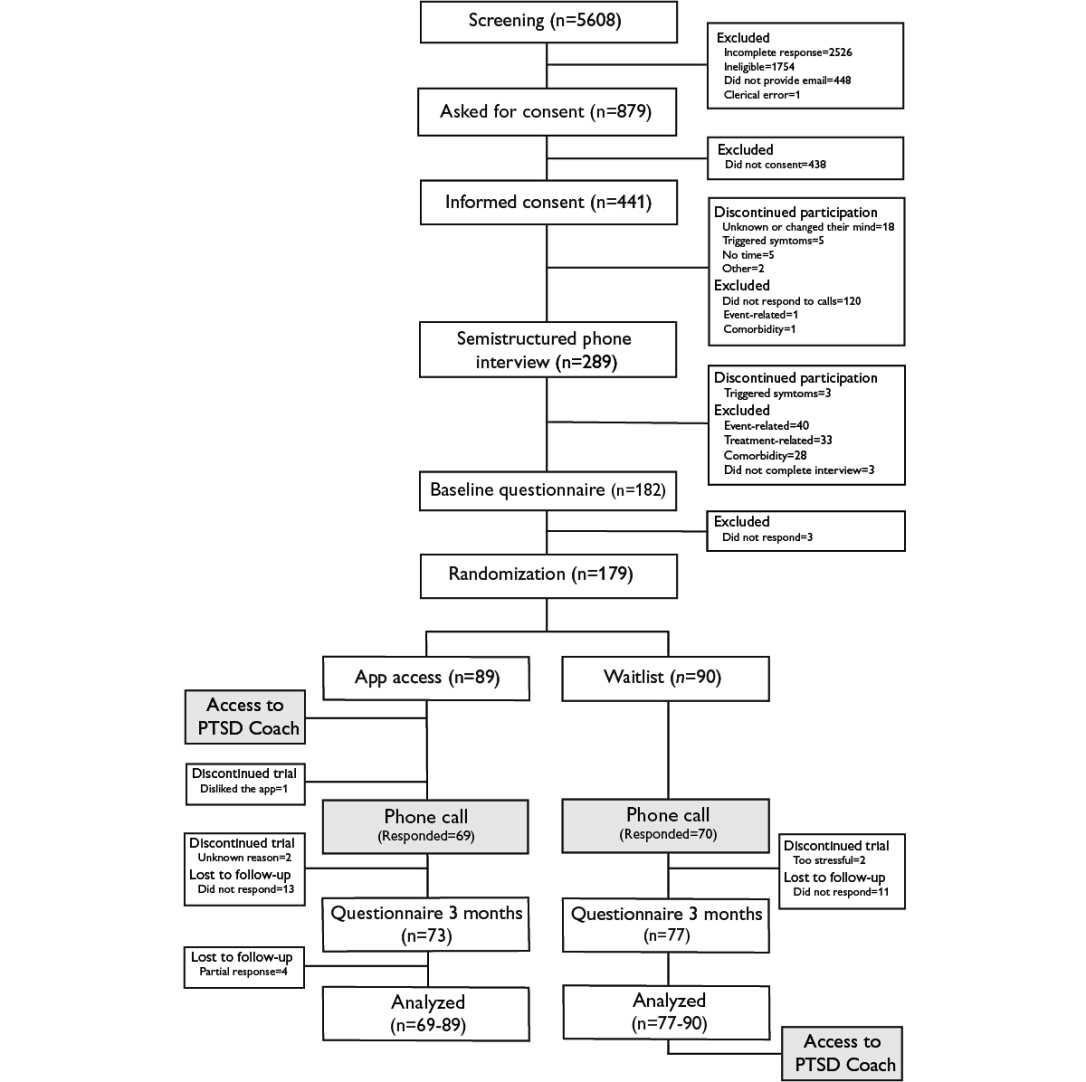
Participant flow and study procedures. PTSD: Posttraumatic Stress Disorder.

A total of 179 adults were included in the study, with 5 participants discontinuing participation after randomization (Figure 1). Using Welch *t* test, we detected no difference in posttraumatic stress between participants who discontinued participation (n=5; mean 50.60, SD 18.82) and those who completed the follow-up (n=150; mean 35.96, SD 15.90; *t*_4.2_=1.72; *P*=.16). Participants who discontinued participation did not differ in baseline depressive symptoms (*t*_4.1_=0.63; *P*=.56) or somatic symptoms (*t*_4.3_=0.35; *P*=.75) compared with completers. However, baseline posttraumatic stress was higher among participants who were lost to follow-up (n=24; mean 42.96, SD 13.54) compared with completers (t_34_=2.29; *P*=.03). Participants who were lost to follow-up did not differ in baseline depressive (t_32_=0.05; *P*=.96) or somatic symptoms (t_30_=0.71; *P*=.48) compared with completers.

### Sample Characteristics

Most participants were women who used their smartphones daily (Table 1). The participants were on average 42.78 years (SD 10.90 years; app access mean 43.42, SD 10.60; waitlist mean 42.15, SD 11.21). We assessed the most severe potentially traumatic event in the past 2 years as perceived by the participants. Positive screening for disorders was assessed using the Mini International Neuropsychiatric Interview (Swedish version 7.0.0).

Most participants had experienced a potentially traumatic event firsthand in the past 2 years (Table 1), with 38 participants (app access: n=21; waitlist: n=17) reporting that their lifetime’s worst potentially traumatic event occurred more than 2 years ago. Several participants (app access=38; waitlist=39) had received previous psychological treatment or counseling after exposure to a potentially traumatic event. The levels of posttraumatic stress, depressive, and somatic symptoms were moderate to high (Table 2), and probable psychiatric disorders were common (Table 1).

**Table 1:** Demographic characteristics and clinician assessment of potentially traumatic events and current psychiatric conditions (N=179).

Characteristic			Total, n (%)	App access (n=89), n (%)	Waitlist (n=90), n (%)
**Gender**					
	Women		164 (91.6)	80 (89.9)	84 (93.3)
	Other^a^		15 (8.4)	9 (10.1)	6 (6.7)
**Civil status**					
	Married or cohabitating		87 (48.6)	43 (48.3)	44 (48.9)
	Single		74 (41.3)	39 (43.8)	35 (38.9)
	Other^b^		18 (10.1)	7 (7.9)	11 (12.2)
**Completed education**					
	University degree		100 (55.9)	54 (60.7)	46 (51.1)
	Senior high school diploma (gymnasium)		59 (33)	26 (29.2)	33 (36.7)
	Other^c^		20 (11.2)	8 (9)	11 (12.2)
**Occupation**					
	Employed full-time or part-time		115 (64.3)	59 (66.3)	56 (62.2)
	Sick leave or unemployed		32 (17.9)	15 (16.9)	17 (18.9)
	Student		18 (10.1)	9 (10.1)	9 (10)
	Other (eg, retired)		14 (7.8)	6 (6.7)	8 (8.9)
**Daily smartphone use**					
	>2 hours		146 (81.6)	76 (85.4)	70 (77.8)
	≤2 hours		33 (18.4)	13 (14.6)	20 (22.2)
	Less than daily		0 (0)	0 (0)	0 (0)
**Exposure proximity**					
	Experienced		99 (55.3)	48 (53.9)	51 (56.7)
	Witnessed		49 (27.4)	21 (23.6)	28 (31.1)
	Was told or repeated exposure		31 (17.3)	20 (22.5)	11 (12.2)
**Event category**					
	Sudden, violent, or unexpected death		44 (24.6)	23 (25.8)	21 (23.3)
	Physical assault or violence		35 (19.5)	17 (19.1)	18 (20)
	Sexual assault or violence		32 (17.9)	14 (15.7)	18 (20)
	Life-threatening illness or injury		26 (14.5)	9 (10.1)	17 (18.9)
	Accident (vehicle or other)		20 (11.2)	14 (15.7)	6 (6.7)
	Other stressful events^d^		22 (12.3)	12 (13.5)	10 (11.1)
**Disorder**					
	**PTSD^e^**		99 (55.3)	51 (57.3)	48 (53.3)
		Subtype depersonalization or derealization	22 (12.3)	11 (12.4)	11 (12.2)
		Subtype delayed onset	12 (6.7)	5 (5.6)	7 (7.8)
	**Suicidality (past month)^f^**		77 (43)	36 (40.4)	41 (45.6)
		Lifetime suicide attempt	43 (24)	23 (25.8)	20 (22.2)
	Depressive episode (current)		57 (31.8)	27 (30.3)	30 (33.3)
	Anxiety disorder^g^		65 (36.3)	32 (36)	33 (36.7)
	Other condition^h^		18 (10.1)	12 (13.5)	6 (6.7)

^a^Men, other, or preferred not to answer.

^b^In a relationship (without cohabitation) or cohabitating with parents or other adults.

^c^Incomplete junior or senior high school diploma or complete vocational degree.

^d^Natural disasters, exposure to explosions, fires, dangerous chemicals, war zones or combat, captivity, or other severe experience.

^e^PTSD: posttraumatic stress disorder.

^f^>0 points on the Mini International Neuropsychiatric Interview suicidality scale, module B.

^g^Panic disorder, agoraphobia, social anxiety disorder, or generalized anxiety disorder.

^h^Bulimia nervosa, binge-eating disorder, obsessive-compulsive disorder, or substance or alcohol abuse (past 12 months).

**Table 2:** Clinical characteristics: observed self-rating of symptoms at baseline and after the intervention (N=179; app access: n=89; waitlist: n=90).

Symptoms and condition		Baseline, mean (SD)	After the intervention, mean (SD)^a^
**Posttraumatic stress (PCL-5^b^)**			
	All	37.31 (15.94)	32.33 (18.44)
	App access	36.44 (16.49)	27.47 (17.61)
	Waitlist	38.17 (15.42)	36.95 (18.13)
**Depressive symptoms (PHQ-9^c^)**			
	All	10.88 (6.68)	10.02 (6.90)
	App access	10.65 (6.79)	8.60 (6.07)
	Waitlist	11.11 (6.59)	11.36 (7.40)
**Somatic symptoms (PHQ-15^d^)**			
	All	12.10 (5.56)	11.49 (5.47)
	App access	11.43 (5.83)	10.48 (5.61)
	Waitlist	12.77 (5.22)	12.44 (5.19)

^a^Attrition at follow-up: n=29; app access: n=16; waitlist: n=13.

^b^PCL-5: Posttraumatic Symptom Checklist for the Diagnostic and Statistical Manual of Mental Disorders, Fifth Edition.

^c^PHQ-9: Patient Health Questionnaire-9, depression.

^d^PHQ-15: Patient Health Questionnaire-15, somatic symptoms.

### Confounding Factors

There was some contamination in both groups during the trial. At follow-up, 4 participants on the waitlist reported having used PTSD Coach (presumably the English version of the app), and 7 participants (app access=4; waitlist=3) reported using a self-management app other than PTSD Coach. We also detected potentially confounding factors—participants started psychological treatment (app access=10; waitlist=10), changed their medication (app access=8; waitlist=10), or started a new medication (app access=10; waitlist=8). Moreover, 26 people sought professional help, such as medical or psychological treatment, related to their trauma (app access=17; waitlist=9) during the intervention period. At follow-up, 17 participants with access to the app stated that they had not used PTSD Coach.

### Primary Outcome: Posttraumatic Stress

**Figure 2:**
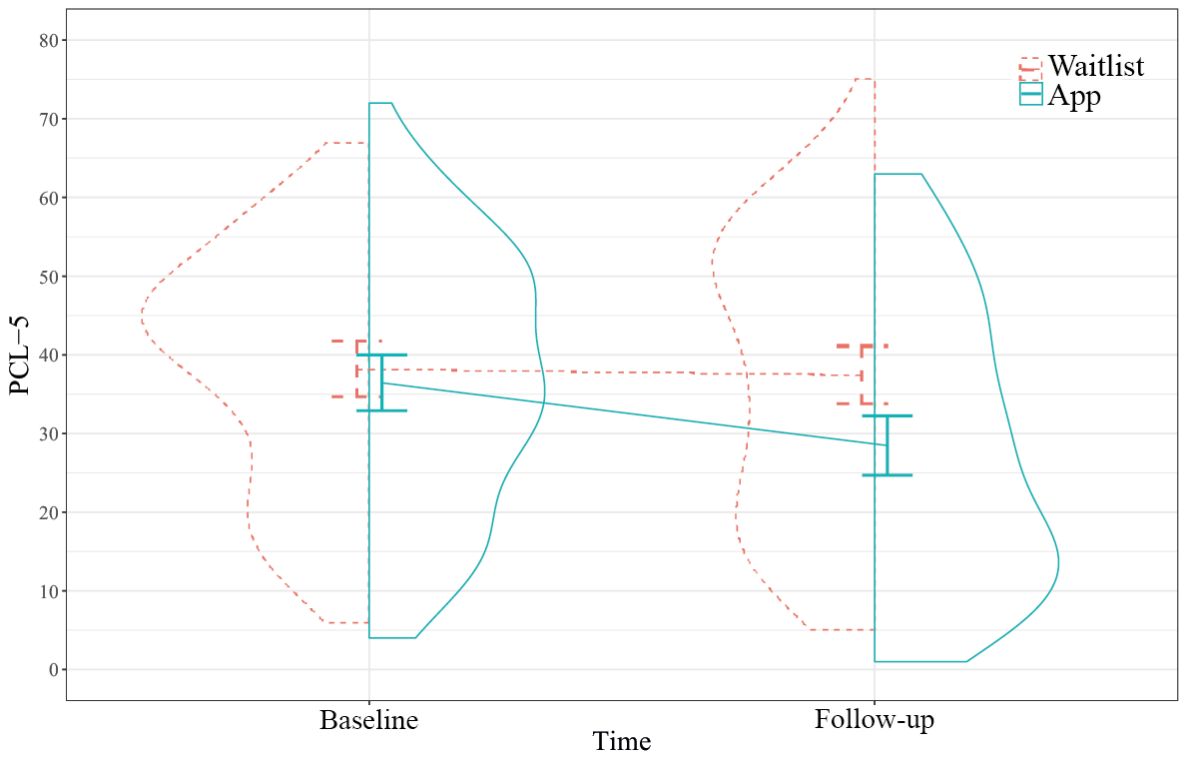
Posttraumatic stress from baseline to follow-up. The panels present pooled, model-based group means and 95% CIs and the distributions of unimputed observations (N=179). PCL-5: Posttraumatic Stress Disorder Checklist for the Diagnostic and Statistical Manual of Mental Disorders, Fifth Edition.

Access to PTSD Coach led to a greater decrease in posttraumatic stress after 3 months compared with the waitlist (Figure 2 and Table 3). The standardized mean difference was small (Cohen *d*=−0.45, 95% CI −0.70 to −0.20). The results from the sensitivity analyses per the protocol and excluding contamination (Multimedia Appendix 4) yielded highly similar results.

Furthermore, we explored the rates of improvement, response, and remission (Table 4). Participants with access to the app were more likely to show clinically significant improvement (*χ*^2^_1,150_=4.62; *P*=.03) and less likely to fulfill the criteria for probable PTSD than participants on the waitlist after 3 months (*χ*^2^_1,150_=7.74; *P*=.005). However, we detected no difference between conditions in remission from probable PTSD (Table 4).

**Table 3:** Parameter estimates, SEs, and CIs for the multiple regression analyses of symptoms, condition, and time (pooled data). We imputed missing data at follow-up (n=29) using predictive mean matching (500 data sets; 10 iterations; N=179).

Outcome and effect		B (SE; 95% CI)	*P* value
**Posttraumatic stress**			
	Intercept	38.17 (1.78; 34.66 to 41.67)	<.001
	Time	−0.74 (1.55; −3.79 to 2.31)	.63
	Condition^a^	−1.73 (2.53; −6.70 to 3.25)	.49
	Condition×time^b^	−7.23 (2.22; −11.60 to −2.85)	.001
**Depressive symptoms**			
	Intercept	11.11 (0.71; 9.71 to 12.51)	<.001
	Time	0.33 (0.71; −1.07 to 1.72)	.65
	Condition^a^	−0.46 (1.01; −2.44 to 1.52)	.65
	Condition×time^b^	−2.34 (1.01; −4.32 to −0.35)	.02
**Somatic symptoms**			
	Intercept	12.77 (0.58; 11.63 to 13.90)	<.001
	Time	−0.34 (0.54; −1.40 to 0.72)	.53
	Condition^a^	−1.33 (0.82; −2.94 to 0.27)	.10
	Condition×time^b^	−0.72 (0.76; −2.22 to 0.79)	.35

^a^0=waitlist, 1=access to the PTSD Coach.

^b^From baseline to follow-up after 3 months.

**Table 4:** Access to PTSD Coach, remission, deterioration, and improvement of posttraumatic stress. App access participants had access to PTSD Coach for 3 months, whereas waitlist participants did not (n=150-179; app access: n=73-89; waitlist: n=77-90).

Outcome	App access		Waitlist		*P* value (*χ*^2^^a^)
	Value, n (%)	Value, N	Value, n (%)	Value, N	
Probable PTSD^b^ at baseline^c^	57 (64)	89	63 (70)	90	.49
Probable PTSD at follow-up^b^	27 (37)	73	47 (61)	77	.005
Remission from PTSD^d^	18 (25)	73	12 (16)	77	.21
Development of PTSD^e^	3 (4)	73	6 (8)	77	.33
Clinically significant improvement^f^	30 (41)	73	18 (23)	77	.03
Clinically significant deterioration^g^	9 (12)	73	20 (26)	77	.06

^a^Development of posttraumatic stress disorder was evaluated with the Fisher exact test.

^b^PTSD: posttraumatic stress disorder.

^c^≥31 points on the Posttraumatic Stress Disorder Checklist for the Diagnostic and Statistical Manual of Mental Disorders, Fifth Edition.

^d^Transitioned from ≥31 to <31 points on PCL-5 from 0 to 3 months.

^e^Transitioned from <31 to ≥31 points on PCL-5 from 0 to 3 months.

^f^≥10 point decrease on Posttraumatic Stress Disorder Checklist for the Diagnostic and Statistical Manual of Mental Disorders, Fifth Edition from 0 to 3 months.

^g^≥10 point increase on the Posttraumatic Stress Disorder Checklist for the Diagnostic and Statistical Manual of Mental Disorders, Fifth Edition from 0 to 3 months.

### Secondary Outcomes

#### Depressive and Somatic Symptoms

**Figure 3:**
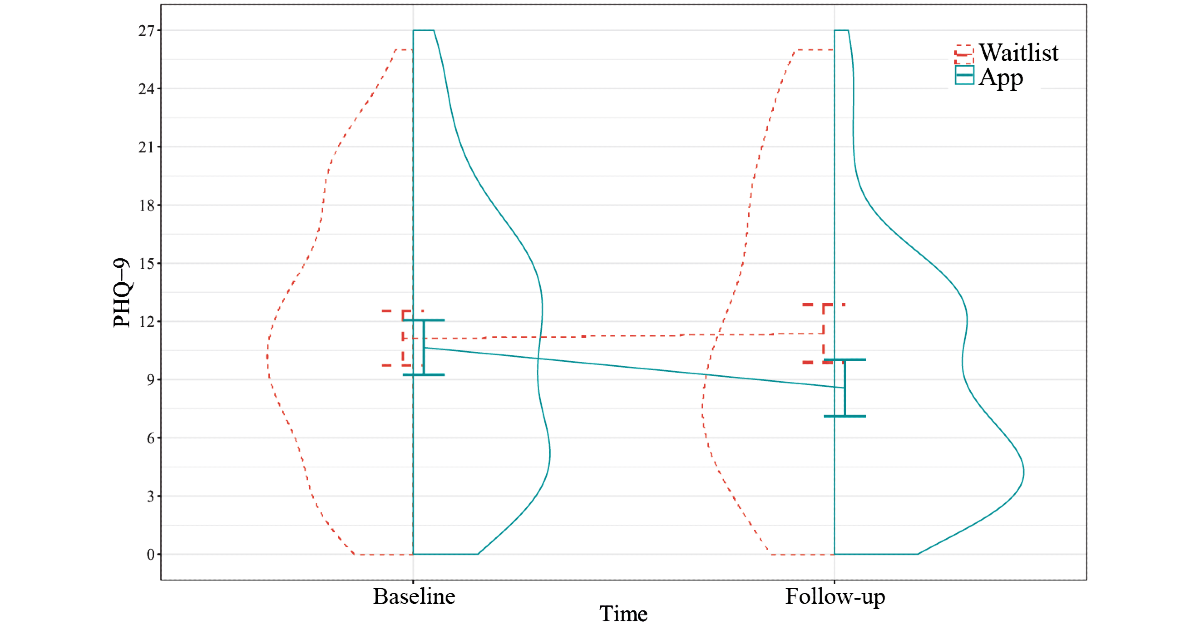
Depressive symptoms from baseline to follow-up. The panels present pooled, model-based group means and 95% CIs and the distributions of unimputed observations (N=179). PHQ-9: Patient Health Questionnaire-9.

**Figure 4:**
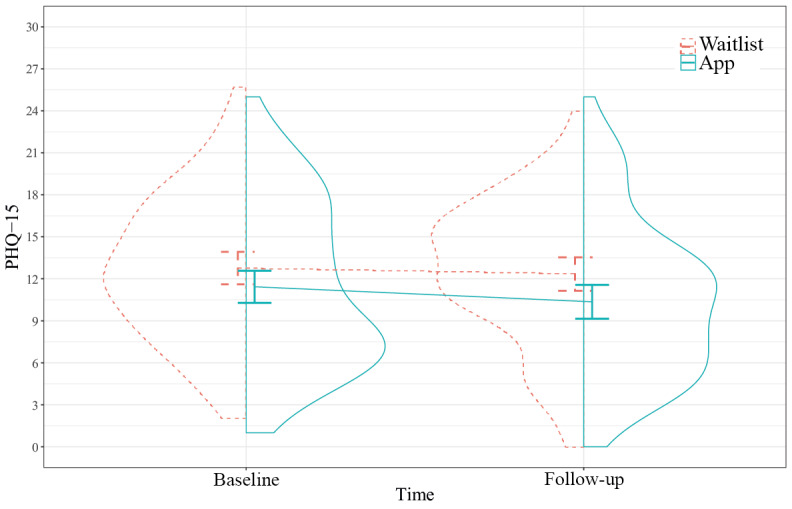
Somatic symptoms from baseline to follow-up. The panels present pooled, model-based group means and 95% CIs and the distributions of unimputed observations (N=179). PHQ-15: Patient Health Questionnaire-15.

Access to PTSD Coach conferred a greater decrease in depressive symptoms after 3 months compared with the waitlist (Figure 3 and Table 3). The standardized mean difference was small (Cohen *d*=−0.35, 95% CI −0.62 to −0.07). The sensitivity analyses per protocol and excluding contamination for depressive symptoms yielded highly similar results (Multimedia Appendix 4). We detected no difference in somatic symptoms based on access to PTSD Coach during 3 months (Figure 4 and Table 3). The standardized mean difference was trivial (Cohen *d*=−0.13, 95% CI −0.38 to 0.12).

#### Helpfulness and Satisfaction

Among participants with access to the app, 4 did not complete the PTSD Coach survey. Average ratings on helpfulness items ranged from 1.29 to 2.03 (Multimedia Appendix 1), which indicates that participants with access to PTSD Coach found the app slightly to moderately helpful. The helpful aspects of PTSD Coach are presented in Multimedia Appendix 5. The average sum score on helpfulness was 23.11 (SD 14.32; n=71). Most participants (50/69, 72%) were moderately or very satisfied with the app (n=69, mean 2.22, SD 1.07).

#### Negative Effects

Only 2 participants with access to the app did not respond to the Negative Effects Questionnaire, whereas 35 reported no negative reactions. In total, 36 people reported 1 to 8 negative reactions that, on average, affected them moderately (mean 2.07, SD 0.86), with an average total sum of 7.44 (SD 6.91; n=36). The most common negative reactions with moderate to extreme impact (Multimedia Appendix 6) were related to the design and evaluation of PTSD Coach, such as unfulfilled expectations on the app (14/71, 20%), its results (10/71, 14%), and perceiving the app as unmotivating (13/71, 18%) or confusing (8/71, 11%). Up to 13% (9/71) of participants experienced negative reactions in the form of psychological symptoms (Multimedia Appendix 2). Moderate to extreme symptom-related reactions included increased stress (7/71, 10%), arousal of distressing memories (6/71, 8%), anxiety (5/71, 7%), and symptom deterioration (5/71, 7%). In contrast, no one reported increased suicidality associated with the PTSD Coach or dependency on the app (Multimedia Appendix 2). Moreover, we detected no difference in symptom deterioration between participants with access to the app and those on the waitlist (Table 4).

## Discussion

### Principal Findings

We conclude that access to PTSD Coach during 3 months decreased posttraumatic stress and depressive symptoms but not somatic symptoms, as compared with a waitlist control. Users perceived PTSD Coach as slightly to moderately helpful and moderately satisfactory. We found no evidence of symptom deterioration among users of PTSD Coach compared with the waitlist, and the most commonly reported negative reactions were related to the evaluation of the app and its design.

The participants’ severity of posttraumatic stress was comparable [10,12] or lower [6,13,14] than the symptom burden in previous studies. Nonetheless, the treatment effect for posttraumatic stress was comparable with the response (range 5-11 points) observed in most previous studies [6,12-14]. Depressive symptoms decreased more than [10,14] or similarly [6,12] to the response in previous studies. Although participants exhibited moderate somatic symptoms, the app primarily targeted psychological distress, which could explain the lack of significant results. In addition, the origin of participants’ mental health and somatic symptoms may be disparate.

The perceived helpfulness and satisfaction were generally lower than the American version of PTSD Coach [5] and greater than in the Swedish pilot study [12]. We speculate that the participants’ lower ratings of helpfulness compared with their satisfaction ratings may, in part, reflect modest expectations of the potential benefits of a smartphone app. Nonetheless, resolving technical issues and further cultural adaptation of the content may improve perceived helpfulness. Similar to participants in clinical trials of psychological treatments [36], approximately half of the intervention participants reported at least one negative reaction associated with using PTSD Coach. Using PTSD Coach may have triggered trauma-related symptoms (eg, stress, anxiety, and distressing memories), which would counteract the app's purpose. However, 87% (62/71) did not experience symptom-related negative reactions related to app use. Furthermore, we detected no differences in self-rated deterioration (clinically significant deterioration or development of probable PTSD) in posttraumatic stress between participants with and without access to PTSD Coach. Instead, access to the PTSD Coach was associated with self-rated response and recovery (ie, more instances of clinically significant improvement and fewer cases of probable PTSD) from posttraumatic stress compared with the waitlist.

### Strengths and Limitations

Our rigorous evaluation of PTSD Coach, an app based on accumulated clinical expertise and research, illuminates the potential positive and negative effects of digital health interventions for posttraumatic stress. The strength of the procedure is that participants on the waitlist and with access to the app received equal attention from the research team. We opted for an inactive waitlist under genuine equipoise as to whether PTSD Coach would be superior, and the results would also, although imperfectly, represent the impact of access to PTSD Coach compared with situations in which professional care may be temporarily unavailable to increase ecological validity; for example, during waitlist for psychological treatment or in the aftermath of mass disaster situations. We did not specifically restrict participants from using sources of support, only other psychological treatments, which would reflect a situation in which access to expert treatment is scarce, but people use other sources of support. Nevertheless, the study design does not permit distinguishing whether PTSD Coach might function as a placebo, which has been discussed as a risk, particularly in smartphone apps [41], as it would entail using a sham or genuinely inactive app as a comparator. In addition, some intervention participants stated that they never used PTSD Coach, which may reflect that they did not receive the intervention. However, some of these participants reported that they had used the app when asked during the first week of the intervention period. To better understand symptom improvement in participants associated with app use, objective app data would be beneficial. We could have ascertained that the users had received the intervention and increased opportunities for gaining app access by sending instructions through multiple channels (eg, email, letter, and text).

We hope that the results of the intervention and negative effects can aid clinicians and users in making balanced and informed decisions about using self-management apps. We know that negative effects occurred, but not to what extent they persisted. Considering that the people lost to follow-up had elevated initial symptoms of posttraumatic stress and depression, negative reactions might be underrepresented. Assessment of negative reactions in the waitlist condition would have enabled a controlled comparison. Nevertheless, the questionnaires would be dissimilar by necessity, as participants on the waitlist did not have access to PTSD Coach.

Another strength is that we used multiple methods of assessment at baseline and assessed both positive and negative outcomes with psychometrically sound instruments. However, the lack of specificity of the symptom measurement could have introduced bias to the promising results: self-ratings on the PCL-5 may not discriminate between posttraumatic stress and depressive symptoms [26]. The PCL-5 has greater sensitivity than specificity for detecting PTSD than the gold standard clinician assessment [26]. Furthermore, the assessment of outcomes is at risk of common method bias; for instance, the clinician screening for PTSD (n=99) differed from self-rated probable PTSD (n=120) at baseline. Multiple forms of assessment (clinician-assessed and self-rated) at follow-up would have improved the validity of the results.

Finally, retention was high, and we did not find evidence of bias associated with missing outcome data. We acknowledge that the predominantly female sample limits the generalizability of the results. Still, the sample was comparable with national estimates regarding marriage or cohabitation (59%) [42], university education (38%-50%) [43], and employment or studies (65%-70%) [44]. We conducted the study in an industrialized, Western society with high rates of smartphone ownership and tax-funded, low-cost mental health care. Similar interventions may need adaptations to promote symptom reduction among other genders or societal contexts.

### Research Implications

In previous studies, the subjectively reported use of PTSD Coach was unrelated to changes in outcomes [6,13]. Therefore, access to PTSD Coach may affect symptoms by moderating processes other than usage frequency. The speculated mechanisms of change by using PTSD Coach include psychoeducation, coping skills, symptom awareness, and social support, which may assist treatment-seeking [6,7,10]. The content of PTSD Coach prompts seeking qualified care, which, if provided, would benefit symptom alleviation. We have limited information regarding adherence to the intervention or use of PTSD Coach during the trial, and future studies would benefit from recording objective use data or contextual information, such as when and where the use of a self-management app successfully mitigates short-term distress. Future research into the mechanisms of change and moderating processes would greatly advance the field and future design of effective mobile self-management interventions for populations in need. We also encourage others to explore the extent and persistence of negative effects concerning psychological or self-management interventions.

### Clinical Implications

Given the observed efficacy, benefits, and harms of PTSD Coach, an unguided self-management app has limited potential to cure PTSD and is unlikely to replace evidence-based psychological treatments for posttraumatic stress when such treatment is warranted. Nevertheless, gaining access to PTSD Coach seems superior to no intervention. Therefore, we believe the results support the feasibility of distributing PTSD Coach, along with other means of support, particularly after events that may temporarily restrict access to support or overwhelm treatment resources, such as pandemics, mass casualty situations, or other disasters. We speculate that access to similar content could boost the skills and knowledge acquired in previous counseling or treatment.

The impact of access to PTSD Coach during 3 months resulted in a slight reduction of posttraumatic stress. Although the average symptom reduction did not indicate a clinically significant change in posttraumatic stress, clinically significant improvement was more common among participants with access to PTSD Coach. We believe that the benefits of disseminating a free, low-intensity intervention for potentially reducing trauma-related distress outweigh the risk of the negative effects we recorded. Nonetheless, we advise that users are informed about the possibility of negative effects to make an informed choice before using apps for mental health. Moreover, to adjust expectations, clinicians could inform users of the extent to which PTSD Coach may be beneficial or helpful. PTSD Coach is a low-risk, helpful, and effective intervention for reducing posttraumatic stress and depressive symptoms. Access to PTSD Coach may complement other psychological, medical, and social interventions for PTSD or provide an attainable first step for survivors of psychological trauma to learn, cope, and begin their road to recovery.

## Appendix

Multimedia Appendix 1 Descriptive statistics of the PTSD Coach survey.

Multimedia Appendix 2 Descriptive statistics of the Negative Effects Questionnaire (PTSD Coach version).

Multimedia Appendix 3 Randomization code in R software.

Multimedia Appendix 4 Parameter estimates, standard errors (SEs), and confidence intervals (CIs) for the multiple regression analysis of symptoms by condition and time (sensitivity analysis).

Multimedia Appendix 5 Percentages of moderate to extremely helpful aspects of the PTSD Coach (n=71). Helpfulness was assessed after 3 months of access to the PTSD Coach. Helpfulness was rated as 0 (not at all), 1 (slightly), 2 (moderately), 3 (very), and 4 (extremely).

Multimedia Appendix 6 Percentages of moderate to extreme negative reactions to the PTSD Coach (n=71). Negative reactions were assessed after 3 months of access to PTSD Coach. The impact of negative reactions was rated as 0 (not at all), 1 (slightly), 2 (moderately), 3 (very), and 4 (extremely). NEQ: Negative Effects Questionnaire.

Multimedia Appendix 7 Consort E-Health Checklist (V.1.6.1).

## Abbreviations

DSM-5: Diagnostic and Statistical Manual of Mental Disorders, Fifth Edition
PCL-5: Posttraumatic Stress Disorder Checklist for the Diagnostic and Statistical Manual of Mental Disorders, Fifth Edition
PHQ: Patient Health Questionnaire
PTSD: posttraumatic stress disorder
RCT: randomized controlled trial
REDCap: Research Electronic Data Capture
