# Correction: Efficacy, Benefits, and Harms of a Self-management App in a Swedish Trauma-Exposed Community Sample (PTSD Coach): Randomized Controlled Trial

**DOI:** 10.2196/45847

**Published:** 2023-02-28

**Authors:** Ida Hensler, Josefin Sveen, Martin Cernvall, Filip K Arnberg

**Affiliations:** 1 National Centre for Disaster Psychiatry Department of Medical Sciences Uppsala University Uppsala Sweden; 2 Psychiatry Department of Medical Sciences Uppsala University Uppsala Sweden

In “Efficacy, Benefits, and Harms of a Self-management App in a Swedish Trauma-Exposed Community Sample (PTSD Coach): Randomized Controlled Trial” (J Med Internet Res 2022;24(3):e31419) the authors noted one error.

In Table 1, the number of participants who screened positive for PTSD in the App Access condition was incorrectly published as "38 (42.7%)." This has been changed to "51 (57.3%)."

The correction will appear in the online version of the paper on the JMIR Publications website on February 28, 2023, together with the publication of this correction notice. Because this was made after submission to PubMed, PubMed Central, and other full-text repositories, the corrected article has also been resubmitted to those repositories.

