# Supplementary material for: Efficacy, Benefits, and Harms of a Self-management App in a Swedish Trauma-Exposed Community Sample (PTSD Coach): Randomized Controlled Trial
Source: J Med Internet Res. 2022 Mar 30;24(3):e31419. doi: 10.2196/31419 (PMC9008528; doi:10.2196/31419)
Supplement: Multimedia Appendix 1 [file jmir_v24i3e31419_app1.docx]

# Multimedia Appendix 1. Descriptive statistics of the PTSD Coach survey

| Item | | *M* | (*SD*) |
| --- | --- | --- | --- |
| 1. | Helping me learn about symptoms of PTSD | 1.86 | (1.21) |
| 2. | Helping me learn about treatments for PTSD | 1.68 | (1.12) |
| 3. | Helping me find effective ways of managing my symptoms | 1.86 | (1.15) |
| 4. | Helping me feel more comfortable in seeking support | 1.57 | (1.29) |
| 5. | Helping me feel like there is something I can do about my PTSD | 1.90 | (1.21) |
| 6. | Helping me track my symptoms | 1.89 | (1.39) |
| 7. | Helping me know when I'm doing better or when I'm doing worse | 1.66 | (1.27) |
| 8. | Increasing my access to additional resources | 1.30 | (1.27) |
| 9. | Providing practical solutions to the problems I experience | 1.66 | (1.30) |
| 10. | Helping me overcome the stigma of seeking mental health services | 1.30 | (1.27) |
| 11. | Helping me better understand what I have been experiencing | 1.54 | (1.23) |
| 12. | Enhancing my knowledge of PTSD | 2.03 | (1.22) |
| 13. | Helping me clarify some of the myths about PTSD | 1.69 | (1.36) |
| 14. | Providing a way for me to talk about what I have been experiencing | 1.29 | (1.17) |
| 15. | Overall, how satisfied are you with the PTSD Coach? | 2.22 | (1.07) |

*n*=71 (item 1-14), *n*=69 (item 15).

Items were rated as 0=Not at all, 1=Slightly, 2=Moderately, 3=Very, 4=Extremely.

PTSD=Posttraumatic Stress Disorder.
