# Supplementary material for: Efficacy, Benefits, and Harms of a Self-management App in a Swedish Trauma-Exposed Community Sample (PTSD Coach): Randomized Controlled Trial
Source: J Med Internet Res. 2022 Mar 30;24(3):e31419. doi: 10.2196/31419 (PMC9008528; doi:10.2196/31419)
Supplement: Multimedia Appendix 2 [file jmir_v24i3e31419_app2.docx]

# Multimedia Appendix 2. Descriptive statistics of the Negative Effects Questionnaire (PTSD Coach version)

|  | Item | Frequency | (%) | *M* | (*SD*) |
| --- | --- | --- | --- | --- | --- |
| 1. | I had more problems with my sleep | 1 | (1) | 0.38 | (1.06) |
| 2. | I felt like I was under more stress | 8 | (11) | 1.31 | (1.45) |
| 3. | I experienced more anxiety | 7 | (10) | 1.00 | (1.25) |
| 4. | I felt more worried | 3 | (4) | 1.00 | (1.50) |
| 5. | I experienced more hopelessness | 3 | (4) | 1.00 | (1.00) |
| 6. | I experienced more unpleasant feelings | 4 | (6) | 0.88 | (1.13) |
| 7. | I felt that the issue I was looking for help with got worse | 5 | (7) | 2.60 | (0.55) |
| 8. | Unpleasant memories resurfaced | 9 | (13) | 1.11 | (1.49) |
| 9. | I became afraid that other people would find out I was using the app | 1 | (1) | 2.00 | . ^b^ |
| 10. | I got thoughts that it would be better if I did not exist anymore or that I should take my own life | 0 | (0) | . ^a^ | . ^b^ |
| 11. | I started feeling ashamed in front of other people because I was using the app | 2 | (3) | 2.00 | (0.00) |
| 12. | I stopped thinking that things could get better | 1 | (1) | 1.00 | (2.00) |
| 13. | I started thinking that the issue I was seeking help for could not be made any better | 2 | (3) | 1.40 | (1.95) |
| 14. | I think that I have developed dependency on the app | 0 | (0) | . ^a^ | . ^b^ |
| 15. | I did not always understand the content of the app | 9 | (13) | 1.50 | (0.85) |
| 16. | I did not always understand the app | 11 | (15) | 1.83 | (0.94) |
| 17. | I did not have confidence in the app | 4 | (6) | 1.80 | (1.30) |
| 18. | I felt that the app did not produce any results | 13 | (18) | 1.81 | (1.47) |
| 19. | I felt that my expectations for the app were not fulfilled | 18 | (25) | 2.10 | (1.17) |
| 20. | I felt that the app was not motivating | 14 | (20) | 2.73 | (1.16) |

The ratings reflect negative reactions that were caused by using PTSD Coach, in contrast to other circumstances, according to participants. The items were rated as 0=Not at all, 1=Slightly, 2=Moderately, 3=Very, 4=Extremely. *N*=71.

^a^ The mean could not be calculated due to insufficient number of responses.

^b^ The standard deviation could not be calculated due to insufficient number of responses.

PTSD=Posttraumatic Stress Disorder.
