# Supplementary material for: Efficacy, Benefits, and Harms of a Self-management App in a Swedish Trauma-Exposed Community Sample (PTSD Coach): Randomized Controlled Trial
Source: J Med Internet Res. 2022 Mar 30;24(3):e31419. doi: 10.2196/31419 (PMC9008528; doi:10.2196/31419)
Supplement: Multimedia Appendix 3 [file jmir_v24i3e31419_app3.docx]

# Multimedia Appendix 3. Randomization code in R software

library(blockrand)

# n=1000 with blocksize=20

d = blockrand (1000, block.sizes=c(10,10))

attach(d)

options(max.print=10000)

#Save to text file

sink("del1.txt")

d[c(1:1000) , 1:4]

sink()
