# Supplementary material for: Efficacy, Benefits, and Harms of a Self-management App in a Swedish Trauma-Exposed Community Sample (PTSD Coach): Randomized Controlled Trial
Source: J Med Internet Res. 2022 Mar 30;24(3):e31419. doi: 10.2196/31419 (PMC9008528; doi:10.2196/31419)
Supplement: Multimedia Appendix 4 [file jmir_v24i3e31419_app4.docx]

# Multimedia Appendix 4. Parameter estimates, standard errors (SEs), and confidence intervals (CIs) for the multiple regression analysis of symptoms by condition and time (sensitivity analysis)

| Analysis | Effect | *B* | *SE* | 95% *CI* | | *P* |
| --- | --- | --- | --- | --- | --- | --- |
|  |  |  |  | Lower | Upper |  |
| Per protocol (*n*=179) | | | | | |  |
| Posttraumatic stress | |  |  |  |  |  |
|  | Intercept | 38.17 | 1.68 | 34.87 | 41.46 | <.001 |
|  | Time | -0.52 | 1.54 | -3.54 | 2.50 | .73 |
|  | Condition ^a^ | -1.73 | 2.39 | -6.41 | 2.95 | .47 |
|  | Condition × Time ^b^ | -7.03 | 2.21 | -11.35 | -2.71 | .002 |
| Depressive symptoms | |  |  |  |  |  |
|  | Intercept | 11.11 | 0.71 | 9.72 | 12.50 | <.001 |
|  | Time | 0.32 | 0.71 | -1.07 | 1.72 | .65 |
|  | Condition ^a^ | -0.46 | 1.00 | -2.43 | 1.51 | .65 |
|  | Condition × Time ^b^ | -2.37 | 1.00 | -4.36 | 0.38 | .02 |
| Without contamination (*n*=169) | | | | | |  |
| Posttraumatic stress | |  |  |  |  |  |
|  | Intercept | 38.94 | 1.74 | 35.50 | 42.38 | <.001 |
|  | Time | -0.47 | 1.55 | -3.54 | 2.60 | .76 |
|  | Condition ^a^ | -2.20 | 2.45 | -7.05 | 2.65 | .37 |
|  | Condition × Time ^b^ | -6.50 | 2.21 | -10.87 | -2.13 | .004 |
| Depressive symptoms | |  |  |  |  |  |
|  | Intercept | 11.40 | 0.73 | 9.96 | 12.85 | <.001 |
|  | Time | 0.39 | 0.74 | -1.08 | 1.85 | .60 |
|  | Condition ^a^ | -0.55 | 1.03 | -2.58 | 1.49 | .60 |
|  | Condition × Time ^b^ | -2.33 | 1.05 | -4.41 | -0.25 | .03 |

Participants who reported contamination (had used a different self-management app or were in the control group and used PTSD Coach, *n*=10) were excluded from uncontaminated models. Missing assessments after 3 months (App access=16, Waitlist=13) were not imputed.

^a^ 0=waitlist, 1=access to PTSD Coach.

^b^ From baseline to follow up after 3 months.
