# Supplementary material for: Efficacy, Benefits, and Harms of a Self-management App in a Swedish Trauma-Exposed Community Sample (PTSD Coach): Randomized Controlled Trial
Source: J Med Internet Res. 2022 Mar 30;24(3):e31419. doi: 10.2196/31419 (PMC9008528; doi:10.2196/31419)
Supplement: Multimedia Appendix 5 [file jmir_v24i3e31419_app5.docx]

# Multimedia Appendix 5. Percentages of moderate to extremely helpful aspects of the PTSD Coach (n=71). Helpfulness was assessed after 3 months of access to the PTSD Coach. Helpfulness was rated as 0 (not at all), 1 (slightly), 2 (moderately), 3 (very), and 4 (extremely)


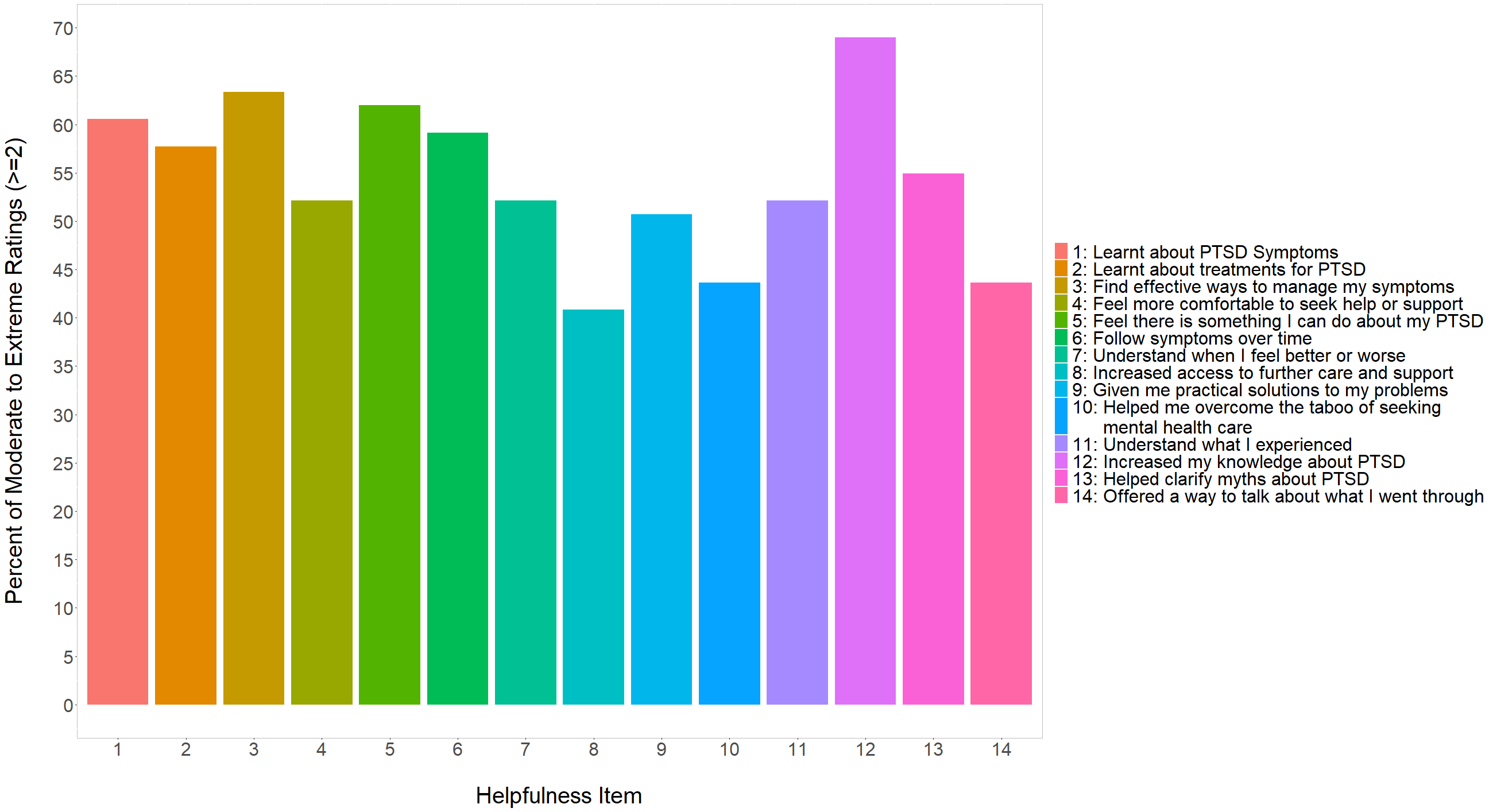


Helpfulness was assessed after 3 months of access to PTSD Coach. *N*=71. Helpfulness was rated as 0=Not at all, 1=Slightly, 2=Moderately, 3=Very, 4=Extremely.

PTSD=Posttraumatic Stress Disorder.
