# Supplementary material for: Efficacy, Benefits, and Harms of a Self-management App in a Swedish Trauma-Exposed Community Sample (PTSD Coach): Randomized Controlled Trial
Source: J Med Internet Res. 2022 Mar 30;24(3):e31419. doi: 10.2196/31419 (PMC9008528; doi:10.2196/31419)
Supplement: Multimedia Appendix 6 [file jmir_v24i3e31419_app6.docx]

# Multimedia Appendix 1. Percentages of moderate to extreme negative reactions to the PTSD Coach (n=71). Negative reactions were assessed after 3 months of access to PTSD Coach. The impact of negative reactions was rated as 0 (not at all), 1 (slightly), 2 (moderately), 3 (very), and 4 (extremely). NEQ: Negative Effects Questionnaire.


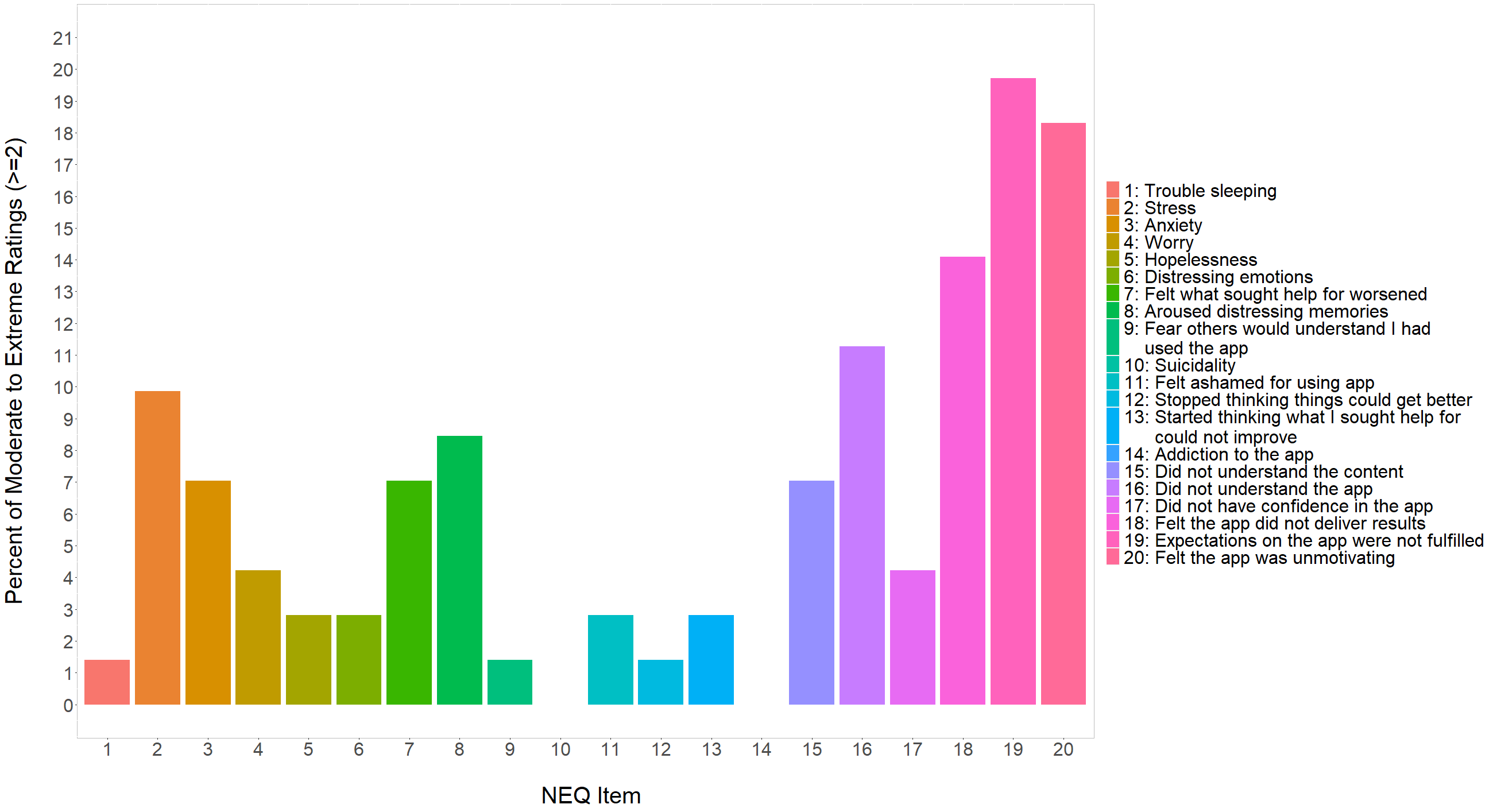


Negative reactions were assessed after 3 months of access to PTSD Coach. *N*=71.

Abbreviation: *NEQ,* Negative Effects Questionnaire. PTSD=Posttraumatic Stress Disorder. Impact of negative reactions were rated as 0=Not at all, 1=Slightly, 2=Moderately, 3=Very, 4=Extremely.
